# Surface evaluations of a nanocomposite after different finishing and polishing systems for anterior and posterior restorations

**DOI:** 10.1002/jemt.23850

**Published:** 2021-06-09

**Authors:** Riccardo Monterubbianesi, Vincenzo Tosco, Giulia Orilisi, Simone Grandini, Giovanna Orsini, Angelo Putignano

**Affiliations:** ^1^ Department of Clinical Sciences and Stomatology Polytechnic University of Marche Ancona Italy; ^2^ Department of Medical Biotechnologies University of Siena Siena Italy

**Keywords:** composite resins, dental finishing, dental polishing, dental restoration, nanocomposite

## Abstract

This study aims to evaluate the effects of different finishing and polishing (F/P) systems on gloss and surface morphology of a new nanocomposite. Thirty discs of Filtek Universal Restorative material (3 M, ESPE) were prepared and divided into six groups (*n* = 5). Group A and B followed F/P protocols for anterior restorations, whereas Group C and D for posterior ones. Group E represented the control (covered by Mylar strip) and Group F represented the nanocomposite placement by means of clinical hand instruments; Groups E and F did not undergo F/P procedures. Among the polished groups, Group B showed the highest values (68.54 ± 7.54 GU), followed by Group A and D (46.87 ± 5.52 GU; 53.76 ± 2.65 GU). Finally, Group C (37.38 ± 4.93 GU) displayed the lowest results. Overall, Group E showed the highest gloss values (93.45 ± 8.27 GU), while Group F presented the lowest ones (1.74 ± 0.64 GU). Surface analysis revealed that Group A, C, and D displayed a smooth surface. Group B showed the lowest irregularities. Group E exhibited the most uniform superficial morphology. On the other hand, Group F displayed the most irregular one. In conclusion, using the tested material, only two protocols achieved appropriate gloss values. Then, clinicians might use the protocols of Group B and Group D, for anterior and posterior restorations, respectively.

## Graphical abstract

The clinician should avoid unpolished surface in resin‐based composite restorations. In finishing and polishing procedures rigid instruments can be used on anterior restorations instead than in posterior ones. On the other hand, for posterior restorations, flexible instruments, which perfectly adapt to the complex occlusal anatomy, should be used.

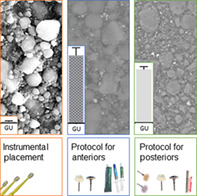

## 1 INTRODUCTION

Nowadays, direct restorations represent a challenge for dental clinicians, due to the high aesthetic demands of patients regarding both anterior and posterior areas. Resin‐based composites (RCs) are considered the gold standard for dental restorations and, in the recent years, thanks to nanotechnology, RCs were improved with nanofillers, thus introducing the so‐called nanocomposites in the market (Demarco et al., 2015). By decreasing the size of the filler, the resulting nanocomposite has ameliorated its physical and aesthetic properties (Cavalcante, Masouras, Watts, Pimenta, & Silikas, 2009; Heintze, Forjanic, Ohmiti, & Rousson, 2010; Moszner & Klapdohr, 2004). The aesthetic outcomes of these materials are strongly influenced by the final surface treatments (Magdy et al., 2017). In this light, accurate finishing and polishing (F/P) procedures are also crucial to enhance the longevity of composite restorations and to achieve a satisfactory surface roughness and gloss (Babina et al., 2020).

Gloss plays an important role in aesthetic dental restorations since the differences in gloss between the restoration and the surrounding enamel are easily detectable by the human eye, even when there is a color match between the restoration and the tooth structure (Rodrigues‐Junior, Chemin, Piaia, & Ferracane, 2015). The gloss retention is the ability of the surface to reflect light and it is related with the amount of light reflected by the particles of the material surface (Kaizer, de Oliveira‐Ogliari, Cenci, Opdam, & Moraes, 2014). Some authors correlated gloss with roughness of the restorations and concluded that a glossy surface corresponds to a smooth surface, which exhibits clinical durability and satisfactory aesthetic appearance (Heintze et al., 2010; Lainović et al., 2014). On the other hand, a rough and irregular restoration surface can be easily affected by superficial stains and plaque accumulation (Takahashi et al., 2013), which often lead to gingival inflammation (Park, Song, Jung, Ahn, & Ferracane, 2012) and secondary caries (Aytac et al., 2016; Dutra, Pereira, Kantorski, Valandro, & Zanatta, 2018). After placing and curing the RCs, clinicians should finish and polish their surface in order to emulate dental anatomy, to refine the occlusion match, the shape as well as the margins of the restoration (Antonson, Yazici, Kilinc, Antonson, & Hardigan, 2011; da Costa, Goncalves, & Ferracane, 2011).

Finishing is defined as the gross contouring or reduction of a restoration to obtain an ideal anatomy, while polishing refers to the reduction of roughness and scratches, which are generated by finishing instruments (Erdemir, Sancakli, & Yildiz, 2012; Yap, Sau, & Lye, 1998). A variety of F/P systems are currently available in the market, including multistep discs, fine and superfine diamond burs, abrasive discs, and diamond, silicon, or aluminium oxide‐impregnated soft rubber cups (Daud et al., 2020; Erdemir et al., 2012; Magdy et al., 2017; Yap et al., 1998). Furthermore, the F/P protocols may include the sequential use of instruments and pastes with a progressive decrease in abrasion (Ehrmann, Medioni, & Brulat‐Bouchard, 2018). Since there are such as numerous commercially available products, dentists should combine different instruments in order to achieve the best results in a specific mouth region (Bansal et al., 2019; Marghalani, 2010). Indeed, the shape of such instruments is detrimental for clinical applicability (Silva et al., 2021). Nevertheless, discrepancies in the scientific literature on these issues and the introduction of a new resin material and new polishing systems have revealed the demand for new research on this topic. Therefore, the aim of this in vitro study was to investigate, by means of scanning electron microscopy (SEM) and the glossmeter, the effect of different F/P sequences of a novel nanocomposite on the gloss retention and the surface morphology. The null hypothesis is that there are no differences in gloss retention and surface morphology of the tested nanocomposite submitted to different F/P protocols, for anterior and posterior restorations.

## 2 MATERIALS AND METHODS

### 2.1 Samples preparation

In this in vitro study, a new high viscosity nanofilled composite (Filtek Universal Restorative 3M, St. Paul, MN) was used and its composition is reported in Table 1. Thirty samples were prepared and divided into 6 groups (*n* = 5), according to the protocols used: Group A, B, C, D, E, and F (Table 2). In Group A, B, C, D, and E, the material was placed into a metal disc mold (3.0 mm in height and 6.0 mm in internal diameter) and covered with a transparent Mylar strip to extrude the excess, thus producing a flat surface and excluding the oxygen inhibition during polymerization. In Group F, the material was placed in the metal disc mold by using the following hand instruments: a micro brush, LM‐Arte Applica (LM‐Dental, Pargas, Finland) and LM‐Arte Condensa (LM‐Dental), in order to simulate the daily placement of the composite by a clinician (Chiodera et al., 2021). Then, all samples were cured for 40 s for each surface, using Elipar DeepCure S light‐curing unit (3M, ESPE, St. Paul, MN), with an irradiance around 1,470 mW/cm^2^ and a spectrum range between 430 and 480 nm. Next, samples received different F/P treatments according to the assigned group (Group A, B,C, and D): Group A and B adopted two protocols, suitable for anterior restorations; Group C and D adopted two protocols suitable for the posterior ones. The exact composition of the materials used for F/P procedures has been reported in Table 3. On the other hand, Group E, was considered as control, and Group F remained unpolished. All samples were stored in dry and dark conditions, at room temperature, during the study. Each F/P procedure was performed using a low‐speed handpiece at 15.000 rpm, with constant and repetitive movements to avoid an excessive heat and formation of surface grooves. All F/P procedures were performed by the same operator to get standard results. After the F/P procedures, all samples were rinsed, ultrasonically cleaned for 5 min and air‐dried, to remove debris on the surface. All samples were subjected to gloss and SEM evaluations.

**TABLE 1:** Composition of tested material

Material	Type	Matrix	Composition	Filler load (Vol%—Wt%)
Filtek Universal Restorative 3M ESPE	Nanofilled	AUDMA, AFM, Diurethane‐DMA, 1,12‐dodecane‐DMA	Nonagglomerated/nonaggregated 20 nm silica filler, a nonagglomerated/nonaggregated 4–11 nm zirconia filler, an aggregated zirconia/silica cluster filler (comprised of 20 nm silica and 4–11 nm zirconia particles), and an ytterbium trifluoride filler consisting of agglomerated 100 nm particles.	58.4–76.5%

Abbreviations: AFM, addition‐fragmentation monomer; AUDMA, urethane dimethacrylate; DMA, dimethacrylate.

**TABLE 2:** Description of the protocols for Group A, B,C, D, E (Control Group), and F (material placed by manual)

Group	Protocol	
A		Sof‐Lex Spiral Wheel Brown
		Sof‐Lex Spiral Wheel Rose + Shiny A
		Sof‐Lex Spiral Wheel Rose + Shiny B
		Sof‐Lex Spiral Wheel Rose + Shiny C
B	Sof‐Lex Discs XT: DO, O, LO, Y	Shiny S + Shiny A
		Shiny S + Shiny B
		Shiny F + Shiny C
C		Diamond flame bur #4
		Multiblade bur #5
		Sof‐Lex Spiral Wheel Brown
		Sof‐Lex Spiral Wheel Rose + Diamond Twist SCO
D	Sof‐Lex Discs XT: DO, O	Diamond flame bur #4
		Multiblade bur #5
		Sof‐Lex Spiral Wheel Brown
		Sof‐Lex Spiral Wheel Rose
		Shiny S + Diamond Twist SCO
		Shiny F + Diamond Twist SCO
E	Hawe Striproll	
F	Microbrush	
	LM‐Arte Applica	
	LM‐Arte Condensa	

Abbreviations: DO, dark orange; LO, light orange; O, orange; Y, yellow.

**TABLE 3:** Composition of the materials used

Material	Manufacturer	Description	Particle size
Diamond bur #4 and Multiblade bur #5 Finishing	Komet (Style kit of Style Italiano)	Low speed flame burs (contouring and smoothing) fine multi blade carbide bur (finishing)	Diamond bur #4‐831‐204‐012 Multiblade bur #5‐H48LUF‐314‐012
Diamond Twist SCO	Premier Dental CO	Super‐charged polishing paste	Diamond paste
Enamel Plus Kit Shiny	Micerium	Shiny A	Diamond paste 3 μm
		Shiny B	Diamond paste 1 μm
		Shiny C	Aluminum oxide particles
		Shiny F	Felt disc
		Shiny S	Hair goat brush
Hawe Striproll	Kerr	Transparent mylar strip	8 mm/0.05 mm
LM‐Arte Applica and LM‐Arte Condensa	LM‐Dental	Instrument set designed for aesthetic layering of composite fillings	
Sof‐Lex Disc XT	3M ESPE	Polyester film, alumina grit, and binder	DO—60 μm
			O—29 μm
			LO—14 μm
			Y—5 μm
Sof‐Lex Spiral Wheels	3M ESPE	Elastomer impregnated with aluminum oxide particles	Finishing‐brow‐fine polishing‐rose‐superfine

Abbreviations: DO, dark orange; LO, light orange; O, orange; Y, yellow.

### 2.2 Gloss measurement

Gloss was determined by a Novo‐Curve Glossmeter, using a reference value of 95 gloss units (GU). Each sample was fixed into a silicone mold to avoid light interferences and was placed in the center of the glossmeter. After calibration of the equipment, for each sample, five measurements were evaluated at 60° of light incidence, according to ISO 2813/2014 and reflection angles, compared to the vertical axis, rotating each time the sample by 45°.

### 2.3 SEM evaluation

SEM observations were carried out using a Zeiss Supra 40 electron microscope. After gloss measurements, samples were assembled in a sample holder and metallized with vacuum precipitation of a gold film on the RC surface. SEM worked at 30 kV and at a running distance of 12 mm. For each sample, three pictures at different magnifications, ×400, ×2,000, and ×8,000 were taken. The obtained micrographs were evaluated descriptively, observing the variations in the morphological surface for all different analyzed groups.

### 2.4 Statistical analysis

Normally distributed data deriving from gloss measurement were presented as mean ± standard deviation (*SD*). Significant differences between experimental groups were determined by means of factorial analysis of variance (one‐way ANOVA), followed by Tukey's multiple comparison test, using the statistical software package Prism6 (GraphPad Software, Inc.). Significance was set at *p* <.05.

## 3 RESULTS

**FIGURE 1:**
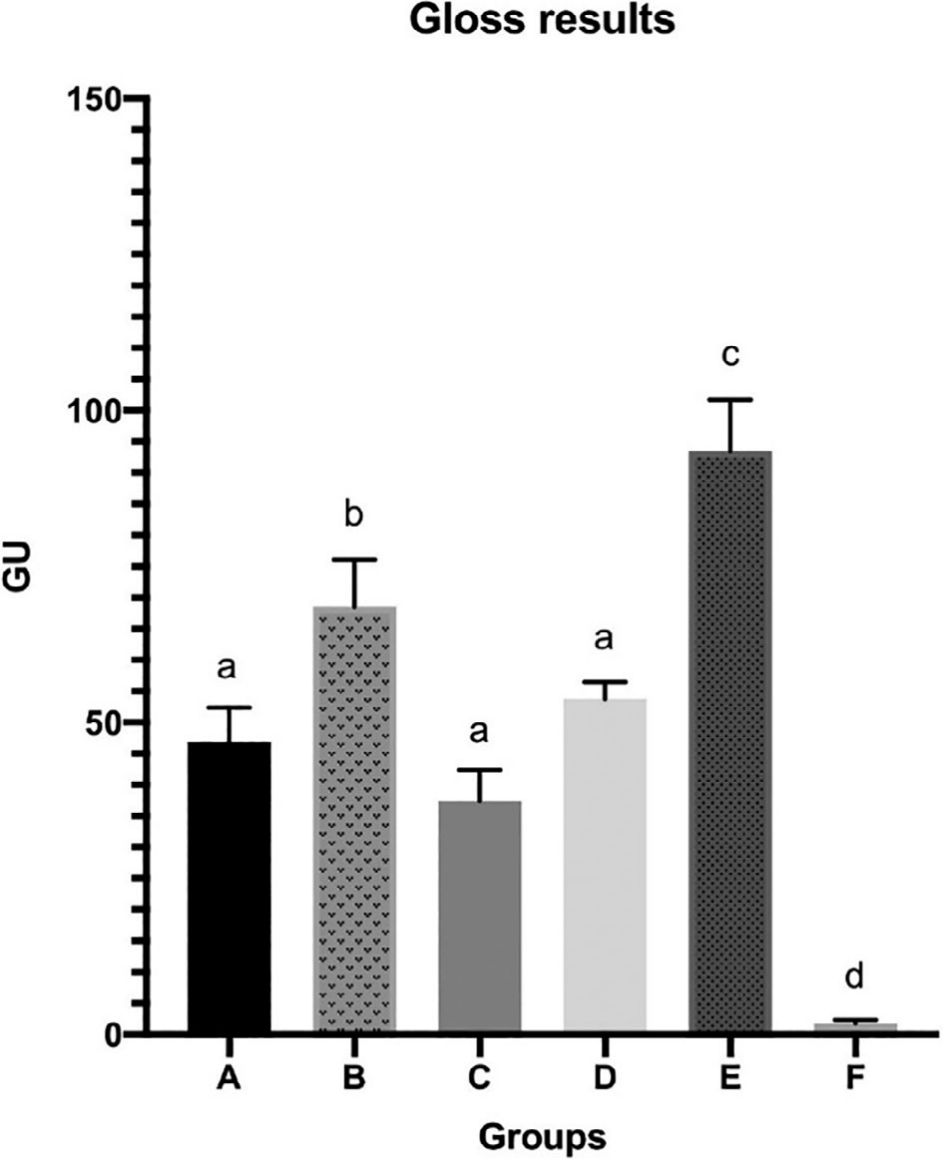
Gloss results. Different superscript letters indicate statistical significance. One‐way ANOVA—Tukey's multiple comparison test, *p* <.05; GU, gloss unit

**FIGURE 2:**
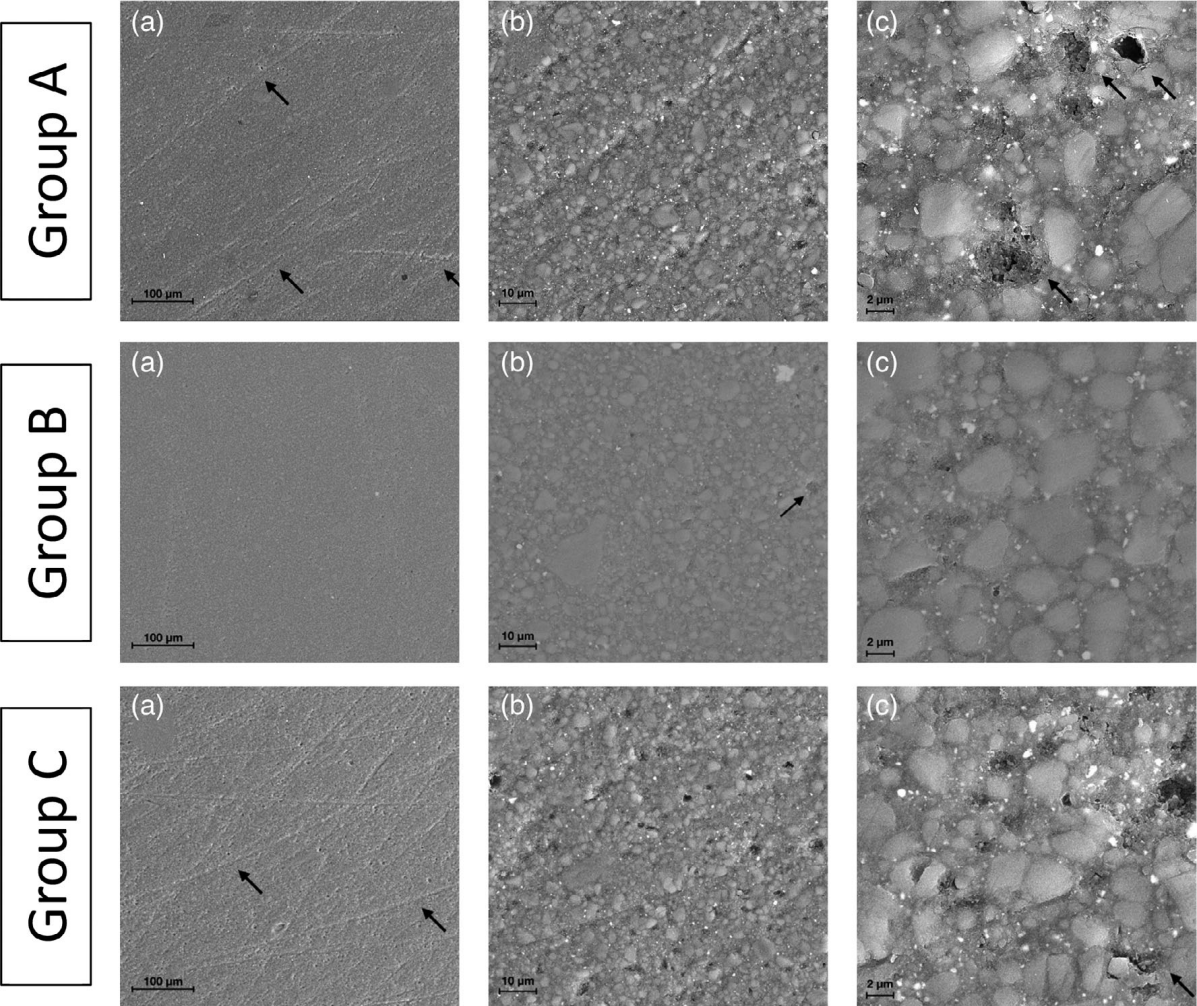
SEM analysis of Group A, B and C after finishing and polishing procedures at different magnifications (a, ×400; b, ×2,000; c, ×8,000). Group A: At low magnification (a, b), the micrographs display a partial regular surface with some scratches (arrows in a). At high magnification (c), the micrograph shows a partial loss of filler particles and resin matrix with the presence of voids (arrows). Group B: At low magnification (a), the micrograph displays a regular surface with the presence of some pores, more evident in b (arrow). At high magnification (c), the micrograph shows a homogenous surface with filler particles and resin matrix. Group C: At low magnification (a, b), micrographs display an irregular surface with scratches and pores (arrows in a). At high magnification (c), the micrograph shows a partial loss of material (arrows)

**FIGURE 3:**
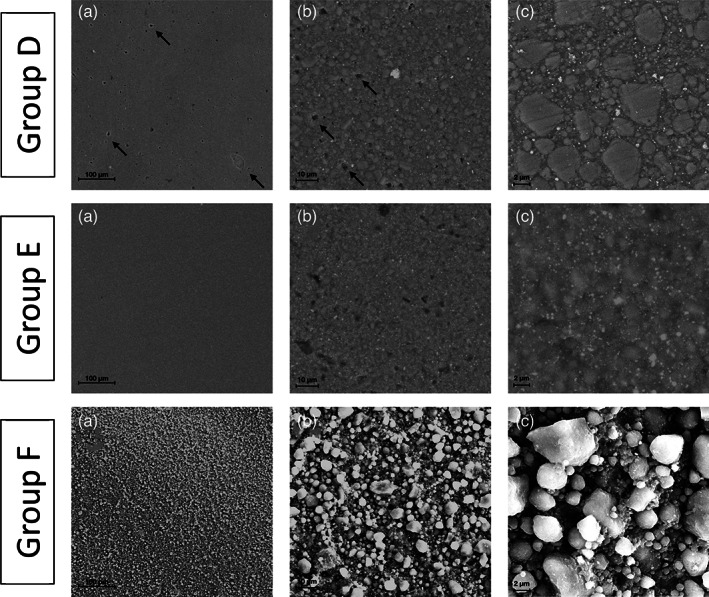
SEM analysis of Group D, E and F after finishing and polishing procedures at different magnifications (a, ×400; b, ×2,000; c, ×8,000). Group D: At low magnification (a), the micrograph displays a partial regular surface with some pores (arrows), more evident in b (arrows). At high magnification (c), the micrograph highlights the resin filler particles with formation of evident scratches. Group E: At low magnification (a), the micrograph displays a regular and uniform surface. However, b shows an irregular surface. At high magnification (c), the micrograph highlights a homogenous surface with resin matrix and filler material. Group F: At low magnification (a, b), the micrographs display an irregular surface. At high magnification (c), the micrograph highlights a surface covered with many spheroidal stone‐like materials of different sizes

The final gloss surface values, according to the applied F/P system, are displayed in Figure 1. Among the groups that underwent finishing and polishing: Group B showed the highest values (68.54 ± 7.54 GU) (*p* <.05) followed by Groups A and D (46.87 ± 5.52 GU; 53.76 ± 2.65 GU, respectively), without statistical differences (*p* >.05), whereas Group C (37.38 ± 4.93 GU) showed the lowest gloss values (*p* <.05). Regarding the specimens not treated with F/P systems, Group E demonstrated the highest gloss values (93.45 ± 8.27 GU), while group F the lowest ones (1.74 ± 0.64 GU) (*p* <.05). SEM analysis was exploited to investigate and compare the surface morphology of each tested group with the not treated ones (Figures 2 and 3). Among the F/P groups, Group A and C highlighted several scratches on the surface (Figure 2), Group B showed the lowest and smallest voids and irregularities (Figure 2, 3) and in Group D many voids were observed (Figure 3). Regarding the unpolished groups, Group E displayed the most uniform morphology surface, presenting no voids and scratches (Figure 3), whereas Group F showed the most irregular morphology. The surface of Group F was composed of isolated spheroidal stone‐like particles of different dimensions, attached on the surface, without resin coverage around them (Figure 3).

## 4 DISCUSSION

The success of RC restorations is correlated to several factors, as aesthetics, biological properties and surface quality (Shimane, Endo, Zheng, Yanagi, & Ohno, 2010). The irregularity of the restoration surface could lead to staining, deterioration, and a reduced efficiency of oral hygiene procedures, hence promoting an increased plaque accumulation and the onset of a secondary caries (Sparabombe et al., 2018). Indeed, F/P procedures are crucial clinical steps to restore the correct anatomical and morphological tooth shape and to provide better optical properties (Antonson et al., 2011; Babina et al., 2020; da Costa et al., 2011). In both anterior and posterior teeth, there are various F/P systems available on the market and the effects of these systems depend on the materials with which they were made and the type of resin composite to be polished (Monterubbianesi et al., 2020). Basically, Cook and Thomas reported, as acceptable, gloss values between 60 and 80 GU (Cook & Thomas, 1990), whereas, according to the American Dental Association (ADA), a typically desired gloss surface corresponds to 40–60 GU (ADA Professional Product Review, 2010).

Our study evaluated the impact of different F/P protocols on a newly developed nanocomposite, simply analyzing its outcomes in terms of gloss values and morphological appearance. The innovation brought by a nanocomposite lies on the fact that it has smaller filler particles that protect the softer resin phase from wear and reduce surface alterations, resulting from loss of such particles (Mitra, Wu, & Holmes, 2003). These advantageous features provide a material with mechanical characteristics suitable for high stress‐bearing restorations with superior aesthetic properties (Heintze, Forjanic, & Rousson, 2006; Mitra et al., 2003; Watanabe, Miyazaki, & Moore, 2006). The tested nanocomposite can be used in both anterior and posterior restorations, therefore, the knowledge of its behavior after different F/P procedures becomes detrimental to achieve the best and reliable results by clinicians.

In summary, the null hypothesis can be rejected. Indeed, in agreement with other studies, Group E provided the glossiest and smoothest surface (Figure 3) (Bansal et al., 2019; Can Say, Yurdagüven, Yaman, & Özer, 2014; Ereifej, Oweis, & Eliades, 2013). Although Group E achieved the highest gloss value, the superficial layer of resin composite in contact with the Mylar strip is more susceptible to wear, besides Group E‐like surface can be achieved only in the interproximal area, neither in occlusal nor buccal surface of restoration. According to the scientific literature, the polished groups have been usually compared only with a control group made using a Mylar strip (Lopes, Monteiro, Mendes, Gonçalves, & Caldeira, 2018; Roeder & Powers, 2004), however, this in vitro study presents as an innovation that the tested Groups were compared even with the surface obtained after using the manual instruments (Group F), without performing F/P, which, indeed, showed the most irregular morphology (Figure 3).

In general, F/P procedures are always required in order to achieve a good results both from a clinical and aesthetic point of view (Endo, Finger, Kanehira, Utterodt, & Komatsu, 2010; Nasoohi, Hoorizad, & Tabatabaei, 2017). Among the finished and polished groups for anterior restorations, Group B provides the best results, with an acceptable gloss value (68.54 ± 7.54 GU). The Group B combines the optimal performance of Sof‐Lex discs together with a progressive decrease in particle size of a diamond paste (Jefferies, 2007). Many authors demonstrated that these flexible discs of aluminum oxide are the best polishing tools for removing the surface irregularities on the anterior teeth, having the ability to cut the filler particles and matrix equally (Bansal et al., 2019; Rodrigues‐Junior et al., 2015). The tested nanocomposite, finished and polished with the protocol of Group B, achieved a gloss value greater than the one reported by the study of Lopes et al. (2018): they evaluated the gloss of another nanocomposite with different F/P protocols, reaching a maximum value of 42 GU. Moreover, in another article, the gloss values of different nanohybrid and micro hybrid resin composites, with different F/P protocols, reached gloss values between 19 and 35 GU (Pala, Tekçe, Tuncer, Serim, & Demirci, 2016). Therefore, the tested nanocomposite, with the proposed protocols, can reach higher GU values than some hybrid resin composites.

Contrary to the front teeth, posterior teeth present a complex occlusal anatomy, thus the flat shape of some F/P instruments risk to flatten the surface of the restoration. In general, the discs are more suitable for F/P procedure on anterior restorations than on posterior ones. However, although the disc shape of Sof‐Lex Spiral Wheel, their flexible rubberized spirals shape design can adapt to nearly every surface of a restoration, both in anterior and posterior reconstructions (da Costa et al., 2011; Pala et al., 2016). Several studies concluded that these elastomer spirals, impregnated with aluminum oxide particles, represent a valid F/P system (Lopes et al., 2018; Moda et al., 2018). Nevertheless, the replication of cusps and grooves of the posterior anatomy still represents a challenge, hence, clinician requires smaller instruments to replicate cusps and grooves of posterior anatomy, like diamond or carbide small burs, in order to remove the excess of RC in posterior restorations (Beltrami et al., 2018). For these reasons, diamond and carbide pointed‐shape burs were included in the protocol of Group C and D, which were set for posterior restorations. Among these groups, Group D achieved an acceptable gloss retention (53.76 ± 2.65 GU): the final association of hair goat brushes and a felt disc, together with a polishing paste, showed an adequate result (Lopes et al., 2018). Indeed, the use of the hair goat brushes and felt equally spreads the diamond paste, allowing a homogeneous reduction in both the resin matrix and the filler particles (Kurt, Cilingir, Bilmenoglu, Topcuoglu, & Kulekci, 2019; Marghalani, 2010; Moda et al., 2018; Tosco et al., 2019).

In addition to the gloss retention evaluation, SEM analysis was carried out to investigate the morphology of the sample surfaces that might not be shown by profilometers (Fuzzi, Zaccheroni, & Vallania, 1996; Moravej‐Salehi, Moravej‐Salehi, & Valian, 2016). In scanning electron micrographs, among the F/P protocols for anterior restorations, Group B displayed a more regular and uniform surface than Group A, confirming the obtained gloss results (Figure 2, 3). On the other hand, Group C and D showed some scratches that made the surface irregular (Figures 2 and 3): the voids and scratches reported by electron micrographs can increase the surface roughness, influencing the gloss, and the surface maintenance. However, the use of pointed burs in those protocols might provoke such irregularities. Although Group F did not show voids and scratches, it displayed the most irregular morphology (Figure 3): the instrumental placement and the sequential curing phase create a surface with spherical agglomerated particles on the surface without resin matrix around them, providing no homogeneous and uneven surface. In a clinical point of view, Group F may represent a surface before the F/P procedures, or even an untreated surface. These findings suggest that composite restorations should be finished and polished as much as possible in order to avoid a Group F‐like surface.

Clinically, other variables might influence the final gloss retention of the surface such as the operator, type of movements, and pressure applied to the instruments (Lins et al., 2016; Nair et al., 2016). Therefore, the F/P procedures were carried out by a single operator to control these variables.

Since only one nanocomposite was evaluated, caution is needed in interpreting the findings and the conclusion of this study should be restricted to the tested material. However, to the best of authors' knowledge, no scientific literature has described the gloss and morphology surface of the tested nanocomposite before and after F/P procedures. Further studies will be planned to evaluate other nanocomposites and different F/P systems, trying to simplify protocols and speed up procedures.

## 5 CONCLUSIONS

Within the limitations of this in vitro study, the following conclusions can be drawn:

1. The best glossy surface is obtained when the tested material is cured under the Mylar strip.
2. After placement of the tested nanocomposite, clinicians should finish and polish the surface in order to not have a Group‐F like surface, full of irregularities.
3. Anterior restorations should be finished and polished following the Group B protocol.
4. Posterior restorations should be finished and polished following the Group D protocol.

## CONFLICT OF INTEREST

The authors declare no potential conflict of interest.

## Data Availability

The data presented in this study are available on request from the corresponding author.
